# Identification of Interactions between Abscisic Acid and Ribulose-1,5-Bisphosphate Carboxylase/Oxygenase

**DOI:** 10.1371/journal.pone.0133033

**Published:** 2015-07-21

**Authors:** Marek M. Galka, Nandhakishore Rajagopalan, Leann M. Buhrow, Ken M. Nelson, Jacek Switala, Adrian J. Cutler, David R. J. Palmer, Peter C. Loewen, Suzanne R. Abrams, Michele C. Loewen

**Affiliations:** 1 National Research Council of Canada, Saskatoon, Saskatchewan, Canada; 2 Department of Chemistry, University of Saskatchewan, Saskatoon, Saskatchewan, Canada; 3 Department of Microbiology, University of Manitoba, Winnipeg, Manitoba, Canada; 4 Department of Biochemistry, University of Saskatchewan, Saskatoon, Saskatchewan, Canada; University of Freiburg, GERMANY

## Abstract

Abscisic acid ((+)-ABA) is a phytohormone involved in the modulation of developmental processes and stress responses in plants. A chemical proteomics approach using an ABA mimetic probe was combined with *in vitro* assays, isothermal titration calorimetry (ITC), x-ray crystallography and *in silico* modelling to identify putative (+)-ABA binding-proteins in crude extracts of *Arabidopsis thaliana*. Ribulose-1,5-bisphosphate carboxylase/oxygenase (Rubisco) was identified as a putative ABA-binding protein. Radiolabelled-binding assays yielded a K_d_ of 47 nM for (+)-ABA binding to spinach Rubisco, which was validated by ITC, and found to be similar to reported and experimentally derived values for the native ribulose-1,5-bisphosphate (RuBP) substrate. Functionally, (+)-ABA caused only weak inhibition of Rubisco catalytic activity (K_i_ of 2.1 mM), but more potent inhibition of Rubisco activation (K_i_ of ~ 130 μM). Comparative structural analysis of Rubisco in the presence of (+)-ABA with RuBP in the active site revealed only a putative low occupancy (+)-ABA binding site on the surface of the large subunit at a location distal from the active site. However, subtle distortions in electron density in the binding pocket and in silico docking support the possibility of a higher affinity (+)-ABA binding site in the RuBP binding pocket. Overall we conclude that (+)-ABA interacts with Rubisco. While the low occupancy (+)-ABA binding site and weak non-competitive inhibition of catalysis may not be relevant, the high affinity site may allow ABA to act as a negative effector of Rubisco activation.

## Introduction

The phytohormone abscisic acid (ABA) modulates plant developmental processes and stress responses [1, 2]. In particular, ABA induction of embryo-specific genes in developing seed leads to accumulation of storage proteins and lipids, desiccation tolerance and inhibition of germination. At the same time, plant responses to environmental stresses, such as drought, high salinity or low temperature result in increased concentrations of ABA which induce expression of stress-related genes leading to, among other events, water retention through stomatal closure.

Ribulose-1,5-bisphosphate carboxylase/oxygnease, or Rubisco, catalyses the carboxylation of D-ribulose-1,5-bisphosphate (RuBP) as the first step in photosynthetic CO_2_ assimilation [3–5]. This is the rate-limiting step in the synthesis of most of the world’s biomass. In spite of its biological importance, Rubisco is an inefficient catalyst, particularly at low CO_2_ concentrations [6]. Turnover rates are in the range of only 0.5–6 s^-1^ across species [7], and this low rate of CO_2_ uptake is further challenged by the ability of the enzyme to also assimilate O_2_ in place of CO_2_, creating a competition modulated by the levels of CO_2_, O_2_ and NADPH. While it remains unclear why such an important enzyme is so slow and multifunctional [8], many molecular aspects are well established. The structure of Rubisco from four higher plants including tobacco, spinach, rice and pea, as well as various algae and bacteria have been reported (reviewed in [9]). All the plant Rubisco structures investigated to date, as well as all but one of the algal and most of the bacterial Rubiscos are hexadecameric, with 8 large (L) subunits and 8 small (S) subunits arranged in two tetramers of L-S dimers with the active site located at an L-L interface, exposed to the solvent through a channel [9, 10]. Rubisco is activated by the addition of CO_2_ (carbamylation) to an active site lysine (residue 201 in the spinach and pea enzymes) which subsequently stabilizes the binding of a Mg^2+^ ion [11]. RuBP then enters the binding cavity and a conformational change involving a single loop closes the RuBP entry channel essentially trapping the substrate [10]. The ensuing carboxylation reaction involves enolization of RuBP, carboxylation of the 2,3-enediolate, hydration of the resulting ketone, carbon-carbon scission and a stereospecific protonation of the resulting carboxylate of one of the product 3-phospho glyceric acids (3PGA [6]).

Many metabolites found in the chloroplast function as modulators of Rubisco activity through interactions in the RuBP binding pocket and at the channel-closing latch site. Inhibitors binding in the catalytic site can block either activation (carbamylation of enzyme) or catalysis (carboxylation of substrate) [12, 13], while activators can bind at both the catalytic and latch sites [14–16]. The complexity of Rubisco regulation is enhanced by the involvement of Rubisco activase which reactivates Rubisco by facilitating the removal of bound inhibitors [17]. Indeed, despite extensive literature, there remains much to be learned about how Rubisco is regulated, especially with respect to its modulation by physiological conditions, including abiotic stress [18, 19].

The modulation of Rubisco activity by environmental stress suggests that Rubisco is regulated as an integral part of basic cellular metabolism. Environmental stressors, including exogenous application of ABA, have classically been believed to inhibit photosynthesis and carbon assimilation through reduction of stomatal conductance [3, 18, 20]. However more recent literature suggests these environmental factors may also modulate these activities through other mechanisms. For example they have now been shown to lead to a decrease in full length Rubisco protein suggestive of increased Rubisco degradation, possibly linked to increased protein oxidation [21–25]. This was confirmed in a recent proteomics study in which added ABA gave rise to an increase in degraded Rubisco fragments [26]. Furthermore, ABA has been shown to mediate these decreases in Rubisco and Rubisco activase content in leaves through induction of vacuolar proteases [27]. Interestingly, Rubisco activase was found to be degraded faster than Rubisco in response to ABA suggesting two-tier methods of Rubisco regulation; reversible inhibition by ligands causes an initial decrease in activity followed by irreversible proteolytic degradation.

**Fig 1:**
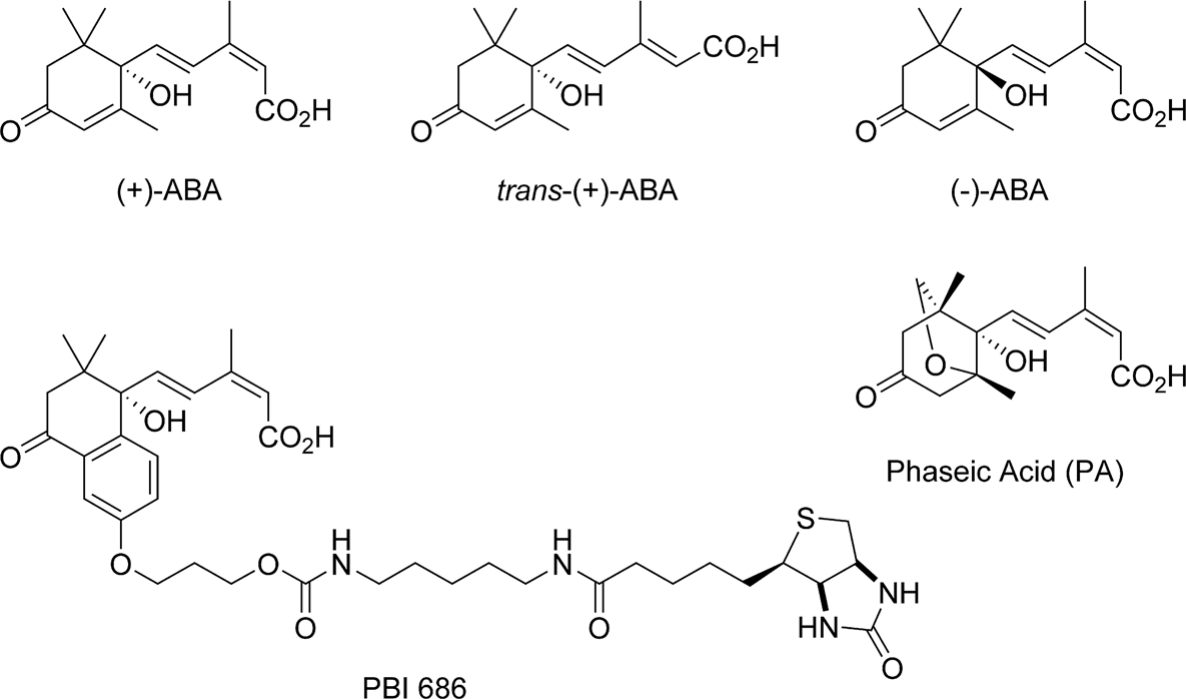
ABA and related ABA analogs. Compounds are labeled accordingly, with (+)-PBI686 representing the photoactive, bioactive ABA-mimetic biotinylated probe used to pull-out putative ABA-binding proteins.

The synthesis, validation and *in vitro* application of an ABA-mimetic photoaffinity-probe, PBI 686 (Fig 1) for the identification of ABA-binding proteins has been previously reported. Targets identified to date have included anti-ABA antibodies, an ABA-8'-hydroxylase, as well as a mitochondrial adenine nucleotide transporter and a human heat shock protein [28–31]. In this report the application of PBI 686 to *Arabidopsis thaliana* (L.) Heynh plant foliar tissue is described, leading to the identification of Rubisco as a putative ABA-binding protein. The binding of ABA to Rubisco was verified by isothermal titration calorimetry and radiolabel binding studies. Attempts to demonstrate a direct functional effect of ABA on Rubisco succeeded in identifying only a weak allosteric inhibition of Rubisco catalytic activity, but a somewhat stronger competitive inhibition of Rubisco activation. While structures derived from Rubisco-RuBP-ABA co-crystals revealed an ABA binding site in proximity to the regulatory latch and Rubisco activase site, incomplete modeling of electron density and computational docking to RuBP free Rubisco support the possibility of ABA binding to the catalytic site. The physiological relevance is discussed.

## Materials and Methods

### Materials

All materials were from Sigma-Aldrich (Oakville, Ontario) unless otherwise indicated. The desalting column (PD-10), HiTrap streptavidin column, Streptavidin-HRP conjugate, ECL biotinylated protein markers and ECLplus Western Blotting Detection Reagents and [^3^H]-(±)-ABA were all obtained from GE biosciences (Baie d’Urfe, Quebec). (+)-ABA was prepared as described previously [32]. PBI686 was synthesized according to Nyangulu et al. [29, 30]. All plotted values are means with standard deviations.

### Preparation of total cell protein extracts from *Arabidopsis thaliana* leaf tissue

Fresh wild type *Arabidopsis* (Columbia, grown in a growth chamber using 12 h photoperiod and temperature of 23–25°C) leaf tissue (40–80 g) was ground with glass beads (500 micron, Aldrich) in 100 mM sodium phosphate buffer at pH 7.6 with 0.33 M sucrose, 40 mM ascorbate and 0.5 mM EDTA and protein inhibitor cocktail buffer (Complete^TM^ Roche, Mannheim, Germany). The homogenate was filtered through cheesecloth and centrifuged at 20,000 g for 10 min. The supernatant was collected and proteins were concentrated by precipitation with 75% ammonium sulfate at 4°C. The precipitated proteins were centrifuged at 5000 *g* for 30 min and re-dissolved in 3–6 mL of phosphate buffer (pH 7.6) with 0.3% non-ionic detergent DHPC (1,2-Diheptanoyl-*sn*-Glycero-3-Phosphocholine; Avanti Polar Lipids, Alabaster, Alabama) to help re-solubilize hydrophobic proteins, for a total protein concentration of approximately 5 mg/mL (concentration determined using SPN^TM^ Protein Assay kit (G-biosciences, St. Louis, MO)). Excess ammonium sulfate was removed by application to a desalting column (PD-10) and proteins concentrated using the protocol supplied with UPPA- Protein-Concentrate^TM^ kit from G-biosciences.

### Chemical proteomic identification of putative ABA binding proteins

The resulting protein extract sample was transferred to Pyrex tubes and mixed with 100 μM PBI686, a photoactivable, biotinylated ABA mimetic probe [8]. The samples were incubated on ice for 1 h and then UV irradiated as described previously using a Hanovia medium pressure mercury lamp for 15 min at room temperature at a distance of 10 cm from the lamp [8, 10]. Samples treated with UV but no PBI686, or those with PBI686 but no UV were used as negative controls. Tagged proteins were enriched by affinity chromatography using a HiTrap streptavidin column (1–3 mL matrix volume) and AKTA^TM^ Explorer FPLC system (Amersham, GE Healthcare, Baie d’Urfe, Quebec). The column was equilibrated with running buffer (20 mM sodium phosphate, 150 mM NaCl, pH 7.5). Protein solutions (5–10 mL; 30–60 mg protein) were injected at a flow rate of 0.3 mL/min, washed with the running buffer for 30–50 column volumes. This was followed by a 10-column volume wash with 0.1 M citric acid (pH 2.0) to remove non-specifically bound proteins. Remaining proteins were eluted with a gradient of 8 M guanidine-HCl at pH 2 (1 mL/min, 30 mL total). Collected fractions were desalted with the PD-10 desalting columns into 20 mM sodium phosphate, 0.15 M NaCl, pH 7.5, concentrated using Amicon^TM^ Ultrafree centrifugal filters (Millipore, Carrigtwohill, Ireland) and again further using the protocol supplied with UPPA- Protein-Concentrate^TM^ kit from G-biosciences. Protein samples were separated by application to 10% acrylamide SDS-PAGE gels. For Western blot analysis, after gel electrophoresis, the proteins were transferred to PVDF membrane for 30 min at 120 V, blocked in a phosphate buffered saline solution (pH 7.5) containing 0.1% v/v Tween (in phosphate buffered saline; PBS-T) with 3% w/v bovine serum albumin and further incubated with Streptavidin-HRP conjugate diluted 1:1000 in PBS-T for 1h at room temperature. ECLplus Western Blotting Detection Reagents were applied to the membrane and luminescence detected using am Amersham Biosciences Storm Scanner (GE HEalthcare, Baie d’Urfe, Quebec) system. ECL biotinylated protein marker was used as molecular weight reference.

Alternatively, gels were stained using FOCUS-FAST silver-stain kit (Genotech, St Louis, MO, USA) according to the manufacturer protocol. Protein bands were excised and placed in a 96-well microtiter plate (Sigma-Aldrich, Oakville ON). The resulting gel pieces were automatically de-stained, reduced with DTT, alkylated with iodoacetamide, and digested with porcine trypsin (sequencing grade, Promega, Madison WI) using a MassPREP protein digest station and recommended procedures (Waters, Manchester, UK). Peptides from tryptic digestion were analyzed using a capLC ternary HPLC system (Waters, Milford, MA) coupled to a Q-ToF Ultima Global (Waters, Manchester, UK). The method used for separation of the peptide digest samples and subsequent analysis using LC-MS/MS and Data Dependent Acquisition (DDA) has been described previously [31]. The LC/MS/MS data were processed using ProteinLynx software (Waters) and searched against databases using MASCOT Daemon and Mascot MS/MS ion search performed on a MASCOT server hosted by NRC (Ottawa, Canada).

### Radiolabelled [^3^H]-(±)-ABA binding assays

An SDS-PAGE analysis of commercially available partially purified spinach Rubisco (Sigma-Aldrich; Catalogue # R8000; specific activity 0.05 units / mg) shows the presence of two, otherwise pure bands that correlate with the expected molecular weights of large (55 kDa) and small (13 kDa) solubilized Rubisco subunits, confirming its purity is sufficient for analysis (S1 Fig). A fresh aliquot of this Rubisco was subsequently dissolved in binding buffer (50 mM bicine, 15 mM MgCl_2_, 20 mM NaCl, pH 7.9) and placed on ice. The total volume for each assay sample was 30 μL. Protein solution (30 μg protein) was mixed with [^3^H]-(±)-ABA (specific activity 37 Ci mmol^-1^ radiolabelled: non-radiolabelled ratio 1.8:1) at the indicated concentrations in the presence and absence of 500 μM (+)-ABA, incubated at RT for 30 minutes. Samples (15 μL) from each reaction were transferred into SPN columns (G-biosciences), to which 100 μL of cold OrgoSol^TM^ buffer (chilled at -20°C) was added. The columns were spun at 10,000 x *g* for 10 s and the wash repeated once. The matrix of each SPN column was then removed and placed in scintillation vials containing Aqasol^TM^ scintillation fluid and soaked for at least 24 hours prior to counting radioactivity using a Beckman Coulter Multi-Purpose Scintillation Counter. Control samples not containing the protein, but treated with [^3^H]-(±)-ABA, were also prepared and values subtracted from experimental samples. Signal corresponding to specific [^3^H]-(±)-ABA binding was calculated from the difference between samples with [^3^H]-(±)-ABA and those containing 1000-fold excess of non-radiolabelled (+)-ABA. Each sample was replicated 5–10 times and readings averaged. Competition was performed as described above, with the concentration of [^3^H]-(±)-ABA at 25 nM in all samples, and non-radiolabeled (+)-ABA added at the indicated concentrations. The same procedure was applied using non-radiolabeled (-)-ABA, PA (phaseic acid), and *trans*-ABA. Values obtained for the same experiment washed with a 10,000 fold excess of unlabelled (±)-ABA were subtracted to account for any non-specific binding.

### Isothermal titration calorimetry

The same, commercially obtained spinach Rubisco (Sigma-Aldrich; catalogue # R8000; S1 Fig; specific activity 0.05 units / mg) was inactivated by dissolving in 10 mM HEPES buffer pH 7.9 containing 100 μM EDTA and dialyzing twice against 2 L of ITC buffer (10 mM HEPES buffer pH 7.9) using a 3,500 MWCO dialysis membrane. A low volume Nano ITC instrument (TA Instruments) with a cell volume of 190 μL was used for this study. Stock solutions of RuBP (100 mM) and (+)-ABA (10 mM) were diluted to the working concentration using ITC buffer. The ITC experiments were performed at 20°C. Rubisco, ligands and buffer were equilibrated at 20°C and degassed before the experiment. A 50 μL syringe was used to deliver 19 injections (2.5 μL each) of the ligands (0.75 mM RuBP or 5 mM (+)-ABA) into the cell containing either ITC buffer or Rubisco (39.42 μM). The first injection of 0.71 μL was excluded from data processing. A stir rate of 200 rpm was used to mix the reactants continuously. The data were processed using the NanoAnalyze software (TA Instruments) using the ‘Independent binding’ model.

### Rubisco activity assays

Commercially available Rubisco from spinach (Sigma-Aldrich; catalogue # R8000; S1 Fig; specific activity 0.05 units / mg) was dissolved in a buffer containing 15 mM MgCl_2_ and 25 mM NaHCO_3_ at pH 8.0 for activation. Each assay (total volume, 1.0 mL) contained 50 mM Bicine (pH 8.0), 15 mM MgCl_2_, 10 mM NaCl, 5 mm DTT, 5 mM phosphocreatine, 5 mM ATP, 5 mM phosphoenolpyruvate, and 25 mM NaHCO_3_. This mixture was prepared before each set of experiments. Five minutes before initiating the reaction with the addition of activated Rubisco, the following were added: RuBP at the indicated concentrations), 0.4 mM NADH, 20 units mL-^-1^ of glyceraldehyde-3-phosphate dehydrogenase, 20 units mL-^-1^ of 3-phosphoglycerate kinase, and 2 units mL^-1^ of creatine phosphokinase. Following the addition of activated Rubisco to a final concentration of 0.3 mg mL^-1^, the NADH concentration was measured spectrophotometrically (λ = 340 nm) at 5 s intervals for 5 min. Absorbance values were converted to NADH concentrations using an extinction coefficient of 6.22 mM^-1^. Reaction rates were evaluated from the slope of absorbance vs. time curves and plotted against concentration of RuBP. Initial velocity (V_i_) vs. substrate concentration [RuBP] plot was constructed using GraphPad Prism^TM^ software, which fit the experimental rate versus concentration data into two different inhibition models (competitive and allosteric). All experiments were performed in the absence of ABA (control) and with (+)-ABA at four concentrations: 1, 2, 5 and 10 mM. For initial rate analysis, toward evaluating Rubisco activation, assays were carried out as described above, but the Rubisco was dissolved in 50 mM bicine (pH 8.0) and dialyzed overnight against the same buffer to ensure the enzyme was in a non-activated state. The non-activated Rubisco was then pre-incubated on ice for at least 10 minutes with or without the indicated concentrations of (+)-ABA, and the reactions initiated and monitored as described above.

### Crystallographic identification of ABA-binding sites

Rubisco was purified from *Pisum sativum* (garden pea; *P*. *sativum*) plants as described previously [33] and co-crystallized with ABA. (+)-ABA (20 mM), dissolved as a sodium salt in water, was added to the protein samples following incubation with 10 mM RuBP, and further incubated on ice for up to 2 hours. Diffraction quality crystals were obtained by the hanging drop vapour diffusion method. Two μl of protein solution was mixed with an equal volume of reservoir solution, the latter containing 10–12% polyethylene glycol 6000 in 100 mM HEPES pH 7.0. The mixed solution drop was then equilibrated against 750 μl of reservoir solution at room temperature. Crystals of approximately 50 x 40 x 20 microns formed within 24–48 hours and were harvested immediately by direct cryopreservation into liquid nitrogen with no additional cryo-preservative. Cryo-preserved crystals were diffracted at the Canadian Light Source, Canadian Macromolecular Crystallography Facility, using the 08ID-1 beam line. The data was processed using MOSFLM and SCALA within the CCP4 software package [34–37]. The space group was determined to be P*2*_1_ *22*_1_ and the unit-cell parameters and processing statistics are included in Table 1. As previously for pea rubisco crystals in the absence of ABA [33], assessment of data quality with phenix.xtriage [38] indicated the possibility of pseudo-merohedral twinning. This twinning, combined with the coincidence of the a and b unit-cell parameters being virtually identical (Table 1), prompted the use of the less common axis choice in the space group. Twinning was further addressed during refinement using the amplitude-based twinning function in REFMAC in the final rounds (twin operator k, h, l). This was consistent with the treatment or RuBP containing pea rubisco crystals reported previously [33]. The structure of *P*. *sativum* Rubisco was solved by molecular replacement based on a single LS unit complex (A and I subunits) from the 8RUC [39] structure of spinach Rubisco, using PHASER through AutoMR in the PHENIX software package [38, 40]. There is one L4S4 unit in the asymmetric unit. The protein structure was refined using a combination of REFMAC (Murshudov et al, 2011) and BUSTER [41] and manual modelling using the molecular graphics program COOT [42]. Water molecules were added automatically with COOT and picked manually. Subunits refined in the asymmetric unit were named A, B, C and D for the L-subunits and S, T, U and V for the S-subunits, as in 4HHH. Refinement statistics are shown in Table 1. The figure was generated using PyMol [43]. The structure has been deposited with PDB ID: 4MKV.

**Table 1:** X-ray data collection and structure refinement statistics. Values in parenthesis are for the highest resolution shell.

***Data collection***	
Space group	P*2*_1_ *22*_1_
Cell dimensions	
a, b, c (Å)	110.44, 110.23, 203.16
α, β, γ	90, 90, 90
Resolution (Å)	48.51–2.15 (2.27–2.15)
No. of unique reflections	128333 (9680)
Completeness (%)	95.6 (97.4)
Rmerge	0.114 (0.446)
<*I/σ(I)*>	6.6 (1.5)
Multiplicity	2.8 (2.7)
***Refinement statistics***	
No. reflections	128282
R factor (%)	16.2
R_free_ (%)	19.7
No. of non-H atoms	18700
No. of waters	1030
rms dev. bonds (Å)	0.010
rms dev. angles (°)	1.13
Av. *B* factor (Å^2^)	30.14
Av. *B* factor (Å^2^)—waters	32.69

### Small molecule docking of abscisic acid to the Rubisco active site

Crystal structures of *Spinacia oleracea* Rubisco in the active, product-bound and non-activated, substrate-bound states (PDB ID: 1AA1 Chain B [44] and PDB ID:1RCX Chain B [45], respectively) were used for ligand binding analysis. A model of the non-activated, open Rubisco state was created by removing the Mg^2+^ ion and carbamylation of Lys201 from the activated, product-bound structure. The abscisic acid ligand structure ((+)-ABA; PDB ID: A8S)) was obtained from the ABA-bound pyrabactin resistance domain of the ABA intracellular receptor (PDB ID: 3ZVU [46]). Small molecule docking was performed using Autodock version 4.2 [47] and its associated graphical user interface AutoDockTools verison 1.5.6. Atomic interaction energy grids were calculated about the Rubisco ligand binding site using the crystallographic glycerate 3-phosphate molecules from the open, product bound structure (PDB ID: 1AA1) and extending 4 Å from any ligand heavy atom for total dimensions of 16.5 Å × 14 Å × 16.5 Å with 0.25 Å grid spacing. The Autodock v4.2 Lamarkian Genetic Algorithm, which combines a global genetic algorithm and a local search, was chosen for the stochastic docking search. Ten search attempts were performed with a grid energy of 1,000; population size of 150; 2,500,000 maximum evaluations; mutation rate of 0.02; and crossover rate of 0.8. The docked ABA conformers were clustered if differing by less than 2.0 Å root mean squared deviation, then ranked based on their predicted ΔG_binding_. Lowest energy representatives from high affinity binding clusters are depicted and discussed herein. PyMol molecular graphics software (Schrödinger, Inc., New York) [43] was used to create the figure.

## Results

### Identification of *A*. *thaliana* (+)-ABA-binding proteins

As reported previously, the probe PBI 686 is suitable for isolation of ABA-binding proteins using a chemical proteomics approach [28–31]. This probe incorporates three important functions: ABA-activity, a biotin purification and immuno-identification handle, and a photoactivatable crosslinking acetophenone moiety (Fig 1).

**Fig 2:**
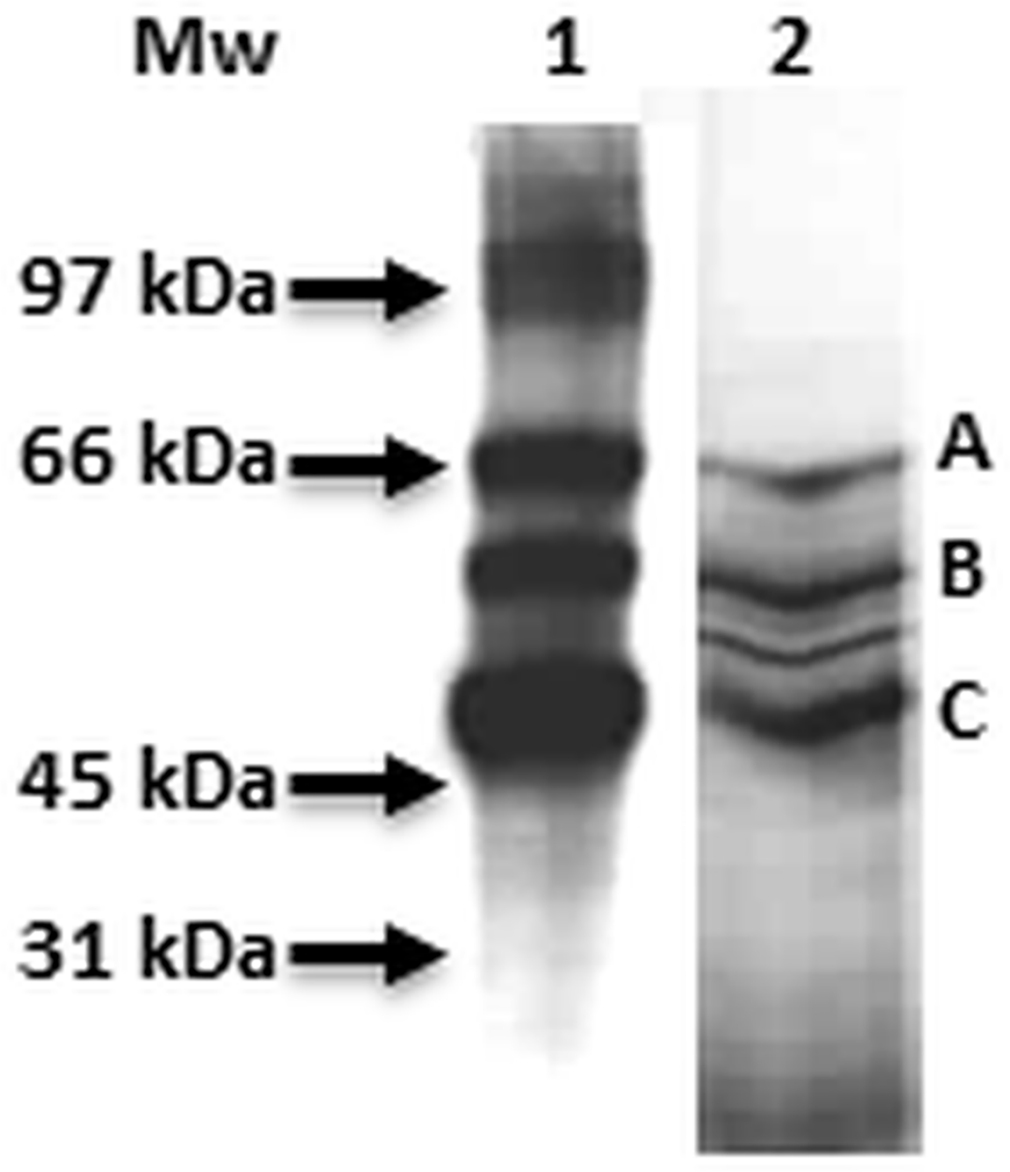
Streptavidin-affinity-enriched PBI686-tagged protein extracts. Total protein extract, photo-cross-linked with PBI686 was enriched using streptavidin—Sepharose affinity chromatography. Eluted proteins were desalted, concentrated and analysed using far-Western blot analysis with a streptavidin—HRP conjugate (lane 1) and silver-staining (lane 2) techniques. Band regions that were excised are indicated with letters A-C. The molecular mass in kDa is indicated.

Using PBI 686, leaf lysates of *Arabidopsis thaliana* were probed. Proteins cross-linked to the probe were then purified by streptavidin-based affinity chromatography, and visualized in Western blots (Fig 2, lane1). Three dominant cross-reacting bands are evident which correspond to 3 of 4 protein bands (Fig 2, lane 2, bands A, B, and C).

Each of the protein bands A, B and C was excised, subjected to tryptic digestion and the resulting peptides analyzed by an LC-MS/MS Q-TOF (quadrupole-time of flight)-coupled Mascot MS/MS ion search (Table 2 and S2 Fig). Band A peptides were matched to three separate thioglucoside-related proteins, including two glucosidases and a glucohydrolase, with similar statistics, which precluded identification of a specific protein but did suggest the possibility of involvement of ABA in glucosinolate metabolism. Bands B and C were matched respectively to three and four different peptides, but the matches with the highest scores were the Rubisco large subunit in band B and the carboxylic ester hydrolase ESM1 in band C. It is noteworthy that band B was also matched to chloroplast ATP synthase beta subunit, with more than 10 matched peptides, highlighting it also likely crosslinked to the probe. This latter finding is consistent with the previous identification of the mitochondrial ATP synthase beta subunit of *Brassica*. *napus* L.cv. Jet Neuf as an ABA binding protein using PBI 686 [28].

**Table 2:** Putative ABA-Binding Proteins Identified from *A*. *thaliana* plant extracts.

**Band**	**Protein Name**	**NCBI-Protein Data**	**Matched Peptides**	**Score**
**A**	Beta-glucosidase Homolog	gi|6651430	46	784
**A**	Thioglucoside Glucohydrolase	gi|984052	31	663
**A**	Thioglucoside	gi|5107821	16	594
**B**	Ribulose-1,5-Bisphosphate Carboxylase/Oxygenase Large Subunit	gi|7525041	82	1153
**B**	Myrosinase-binding Protein Homolog	gi|2997767	7	444
**B**	ATP synthase CF1 beta subunit	gi|7525040	10	286
**C**	Carboxylic Ester Hydrolase ESM1 MODIFIER 1	gi|15231805	69	1171
**C**	Putative Myrosinase-associated Protein	gi|18404748	13	426
**C**	ZW9	gi|18406229	13	412
**C**	Cruciferin 3	gi|15235321	11	403

### (+)-ABA Binds to Rubisco with high affinity

**Fig 3:**
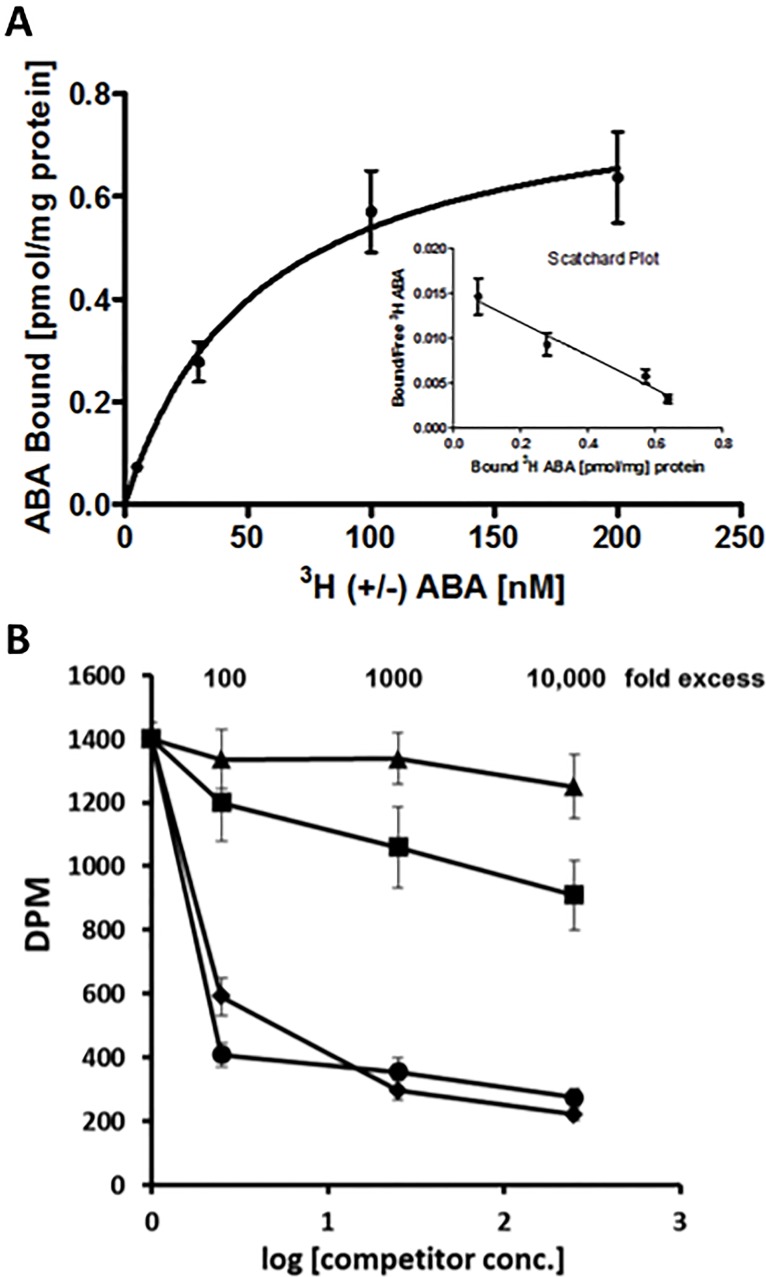
ABA binds to spinach Rubisco with high affinity. A) Saturation curve and Scatchard plot representing specific binding of [^3^H]-(±)-ABA, as a difference between total and non-specific binding signal. B) Competition Binding Assay. Displacement of 25 nM (±) [^3^H]ABA by non-radiolabeled (+) ABA (diamonds), (-) ABA (circles), PA (squares) and trans-(+) ABA (triangle) at the indicated concentrations.

In light of the relevance of Rubisco to plant metabolism, and the high scores obtained in the MS analysis, the interaction of Rubisco with ABA was selected for further characterization. Using a classical radiolabelled [^3^H]-(+/-)-ABA binding assay, previously established and applied to other ABA-binding proteins in our lab [31], a *K*_D_ of 47.0 nM was obtained (Fig 3A) for ABA binding to non-activated Rubisco, suggesting a relatively high affinity interaction. This value is similar, to that obtained previously for RuBP binding to non-activated Rubisco by radiolabelled binding assay (*K*_D_ = 21 nM) [13]. Binding specificity was then assessed by challenging the [^3^H] (±)-ABA binding with non-radiolabeled (+)-ABA, (-)-ABA, phaseic acid (PA) and *trans*- (+)-ABA. (-)-ABA and (+)-ABA were equally effective as competitors, whereas PA and t*rans*-(+)-ABA were ineffective as competitors (Fig 3B). Together these data validate the initial pull-down interaction and suggest a strong and selective binding interaction between ABA and activated Rubisco.

**Fig 4:**
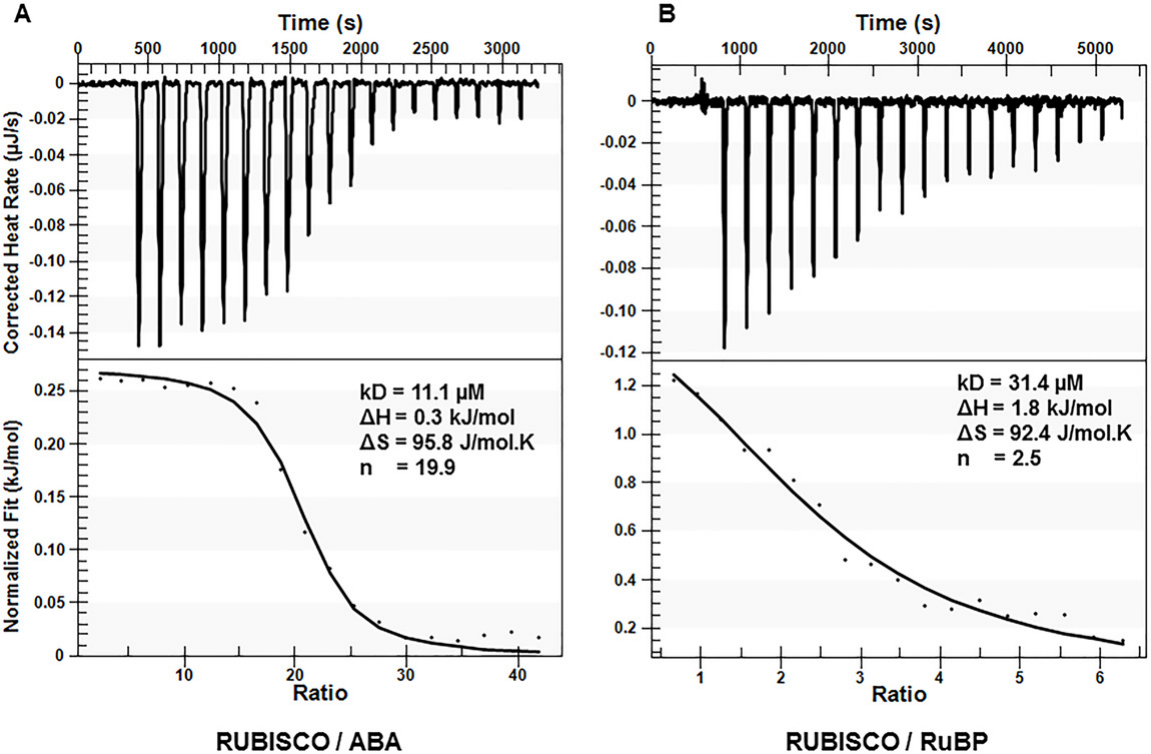
Isothermal titration calorimetry analysis of spinach Rubisco-ligand interactions. Raw ITC data of sequential injections of (A) RuBP (0.75 mM) and (B) (+)-ABA (5 mM) titrated against spinach Rubisco (39.42 μM). The processed fit is shown below each binding isotherm. The binding parameters are shown in the inset. The ligands were titrated until saturation was reached showing a specific binding interaction. The data were fitted using the ‘Independent Binding’ model in the NanoAnalyze software and the best fit is represented by the black line.

Isothermal titration calorimetry (ITC) experiments were subsequently carried out to further validate the binding of ABA to Rubisco. In this system, the analysis yielded a K_d_ of 11.1 μM (Fig 4A) for the spinach Rubisco-ABA interaction, suggesting somewhat lower affinity than that detected by radiolabelling assay. However, this ITC-derived value is similar to that which we previously obtained for ABA binding to the ABA-receptor (PYL5) again by ITC, with a *K*_d_ of 1.2 μM [48]. Interestingly, the ABA-Rubisco binding study yielded a high stoichiometry (n) of 19.9, suggesting that this K_d_ for ABA may represent a mixture of high and low affinity binding sites, which might in part account for the lower affinity detected by this method.

In order to further calibrate these ITC findings, the binding affinity of the enzyme’s natural substrate RuBP was subsequently determined by ITC, yielding a K_d_ of 31.4 μM and a stoichiometry (n) of 2.5 (Fig 4B). Again similar to those obtained for ABA. Interestingly, these values for RuBP are somewhat at odds with those reported previously, *K*_d_ 0.3 μM and n = 3.6, using the same ITC method for non-activated Rubisco [49]. This discrepancy with the literature may be related to the lower specific enzyme activity, 0.05 units / mg reported for the commercially available Rubisco used herein, compared to 1 unit / mg in the previous report [49], it may also be in part related to incomplete inactivation of the enzyme prior to analysis, as the binding affinity of RuBP is actually much lower for activated Rubisco than non-activated (by a factor of ~ 1000) [13].

Regardless, having used the same Rubisco for all binding analyses herein, this data allows a comparison of the interactions of RuBP and ABA with Rubisco, highlighting that ABA does interact with the enzyme with an affinity similar to that of the native substrate. Overall, while these data support the existence of a Rubisco-ABA interaction, the actual affinity and binding stoichiometry remain to be confirmed.

### (+)-ABA inhibits Rubisco

**Fig 5:**
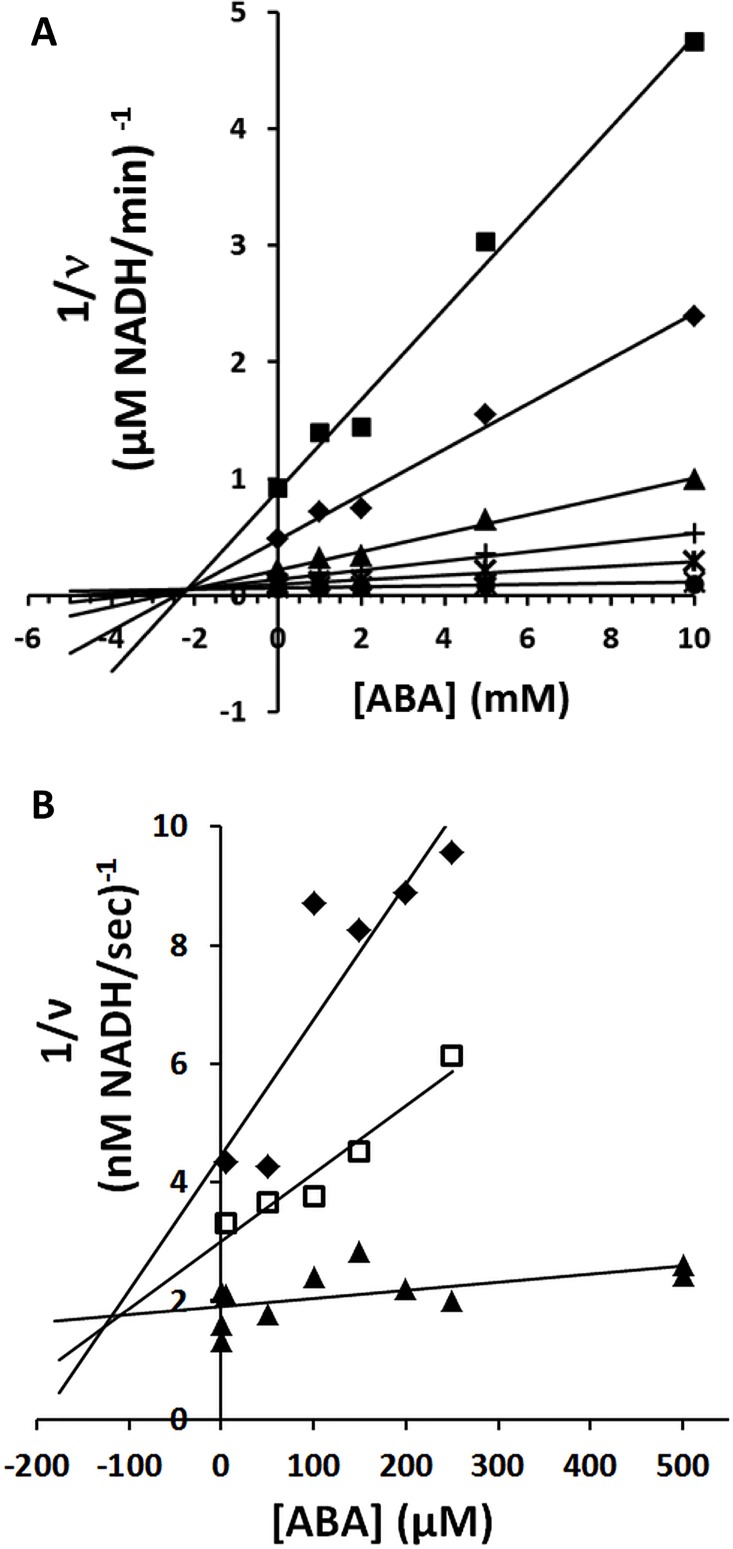
ABA is an inhibitor of spinach Rubisco. A) ABA is a weak non-competitive inhibitor of Rubisco catalytic (carboxylation) activity. Dixon plot showing effect of various concentrations of (+)-ABA on Rubisco catalytic activity in the presence of 10 μM (squares), 20 μM (diamonds), 50 μM (triangles), 100 μM (+), 200 μM (stars), 800 μM (X), 1200 μM (circle) RuBP substrate. The positioning of the intercept close to the x-axis suggests a non-competitive inhibition, with an estimated K_i_ of about 2.1 mM. B) ABA is a competitive inhibitor of Rubisco activation (carbamylation). Dixon plot showing effect of various concentrations of (+)-ABA on Rubisco activation in the presence of 1 mM (black diamonds), 7 mM (open squares) and 20 mM (closed triangles) of the activation substrate NaHCO_3_. The intercept suggests at least partially competitive inhibition with an approximately K_i_ of about 130 μM.

To investigate the biochemical significance of ABA binding to Rubisco, the enzyme’s relative catalytic activity (i.e. carboxylation of RuBP) was compared in the presence and absence of ABA using a coupled reaction assay of pre-activate Rubisco, 3-phosphoglycerate kinase and glyceraldehyde-3-phosphate dehydrogenase in which increasing NADH production is monitored [50]. Initial experiments indicated inhibition of Rubisco catalytic activity by (+)-ABA, but showed no effect of (+)-ABA on control reaction samples lacking Rubisco and RuBP. Kinetic evaluation by Dixon analysis yielded a *K*_i_ of 2.1 mM for inhibition of Rubisco catalysis by (+)-ABA (Fig 5A), suggesting the inhibitory effect here is very weak.

Subsequently, the effect of (+)-ABA on Rubisco carbamylation (i.e. enzyme activation) was investigated, using the coupled reaction described above, except that the Rubisco was not pre-activated and rather pre-incubated with or without (+)-ABA. In this way the activation step is reflected in the initial rate of catalysis measured immediately following simultaneous addition of the activating/catalytic reaction agents [51, 52]. As the enzyme becomes fully activated, an equilibrium rate is achieved. An inhibitory effect of (+)-ABA on activation (initial rate), but not catalysis (equilibrium rate) was observed (S3 Fig). Further kinetic evaluation of this effect by Dixon analysis [53], suggests at least a partially competitive inhibition of Rubisco activation (Fig 5B). The *K*_i_ estimated from this plot for (+)-ABA is approximately 130 μM for Rubisco activation, more than 10-fold more potent than the effect of (+)-ABA on Rubisco catalysis.

Overall, while these data support the inhibition of Rubisco functionalities by ABA, the actual inhibition constants remain to be confirmed.

### Co-crystallization of RuBP-bound Rubisco with (+)-ABA

To identify ABA binding site(s) on Rubisco, crystals of non-activated Rubisco from *Pisum sativum* were grown as described previously (PDB ID: 4HHH, [33]), but in the presence of 20 mM (+)-ABA and in the presence and absence of RuBP. Unfortunately, the absence of RuBP precluded the formation of diffraction quality crystals and thus only a substrate-bound (+)-ABA co-crystal structure is reported. X-ray diffraction data to 2.15 Å (Table 1) were collected and the Rubisco-RuBP-ABA structure refined to 2.15 Å (PDB ID: 4MKV). Like pea Rubisco without ABA [33], the electron density maps define the main chain and side chain atoms of the asymmetric unit comprised of 4 L and 4 S subunits along with 4 RuBP ligands. Because the enzyme was not activated prior to crystallization, the active site lysine is not carbamylated and Mg^2+^ is not present.

**Fig 6:**
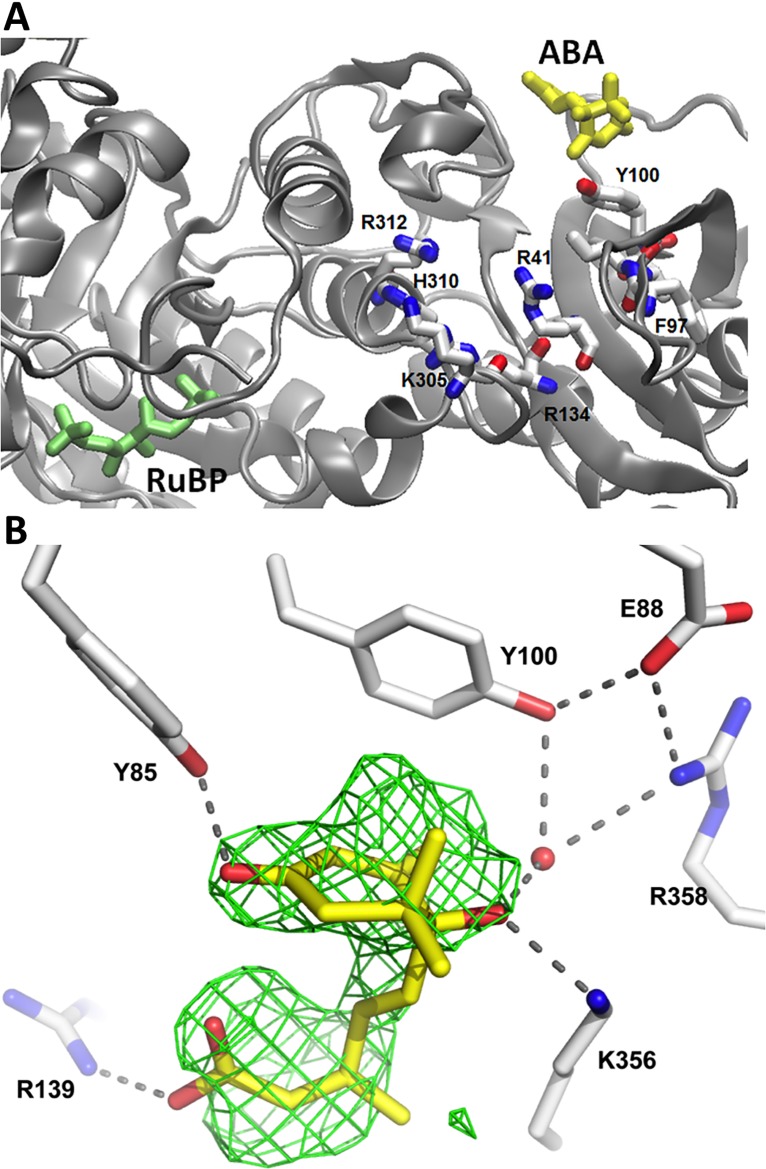
Co-crystallography of RuBP-bound pea Rubisco with ABA. A) L-Subunit B showing (+)-ABA (in yellow) bound to a surface cleft adjacent to Y100. While distal from the RuBP (in green) binding site (~ 30 Å away), this site is in closer proximity to the positively charged regulatory latch region comprised of residues R41, R134, K305, H310, R312 and involves residues that immediately precede and follow the 90–97 loop region implicated in binding to Rubisco activase. B) The omit F_o_-F_c_ electron density maps represented by the green hatching at ~ 2 sigma, were calculated without ABA included in the model. (+)-ABA (in yellow with red oxygens) is shown, bound to the A L-subunit. The terminal carboxylate is shown anchored to R139; the ketone forms a short H-bond with Y85; and the hydroxyl group at the chiral center forms a H-bond with K356. The hydroxyl group also has a weak 3.3 Å interaction with a water that is part of an H-bond and ionic matrix involving the side chains of Y100, E88, R358 and Y363.

To locate possible ABA-binding sites, the *F*_o_−*F*_c_ maps from the ABA-containing crystal were visually searched for regions of unassigned density. In comparison to non-ABA containing crystals, unique density was identified in two L subunits adjacent to Tyr100 and review of the same regions in the other two L subunits confirmed smaller, but visible changes compared to the non-ABA containing maps. The binding sites are similar in each L subunit and are situated in an exterior crevice, remote from any dimer interface and more than 30 Å away from the catalytic site (Fig 6A). Perhaps of significance, the site is close to the regulatory latch region and involves residues that immediately precede and follow the 90–97 loop region implicated in binding to Rubisco activase. (+)-ABA was ultimately best refined into this density in a conformation that is distinct from both the solution or crystal structures of ABA [54–56], as well as that observed in recent structures of ABA bound to its receptor(s) [57]. The cyclohexanone ring and aliphatic tail are oriented about the 4’C– 5C bond such that the carboxylate is situated only 4 Å from the ketone carbonyl (Fig 6B). All three of the polar functional groups of ABA make favorable interactions with the protein. The terminal carboxylate is anchored through an ionic 3.0 Å interaction with the side chain of Arg139 (Fig 6B); the ketone forms a strong 2.7 Å H-bond with the phenolic-OH of Tyr85; and the hydroxyl group at the stereogenic center forms a 2.6 Å H-bond with the side chain of Lys356. The hydroxyl group also has a weak 3.3 Å interaction with a water molecule that is part of an H-bond and ionic matrix involving the side chains of Tyr100, Glu88, Arg358 and the main chain carbonyl of Tyr363.

It must be noted, however, that despite the obvious attributes of a good binding site and the clear presence of unique density in subunits A and B, the density is really only obvious in two of the four subunits, the fit of ABA to the density is not perfect and its B-factors are high compared to surrounding protein suggesting disorder in parts of the ligand. Refinement with 50% occupancy of ABA in the model produced strong F_o_-F_c_ density, highlighting the partial occupancy, but with continued disorder in certain regions of the ligand. Thus the possibility that the unique density is arising from a different molecule, or that it is simply an artifact cannot be strictly eliminated at this time. That said, even if the density does represent ABA, this structural data is inconsistent with the high affinity binding observed by other methods herein. Thus this structural data would only correlate with the weak and non-competitive inhibition of the enzyme’s carboxylation activity (Fig 5A; discussed below), such that the overall relevance of this putative surface ABA-binding site is questionable.

In light of no obvious high affinity ABA-binding site outside of the binding pocket of Rubisco, closer inspection of the catalytic substrate-binding site was undertaken. Here a slight distortion (elongation) of the electron density in the region of the hydroxyl on C4 of the RuBP molecule, as compared to that observed in the absence of ABA. While this distortion might be accounted for by low occupancy substitution of RuBP with (+)-ABA, the evidence here is again inconclusive due to the overlap. While this possibility is further probed through *in silico* docking studies (below), any conclusive demonstration of ABA binding to the active site awaits ABA-bound RuBP-free Rubisco crystals.

Finally, superimposition of the ABA-Rubisco structure (PDB ID: 4MKV) with that of the non-ABA Rubisco structure (PDB ID: 4HHH) yielded a root mean square deviation (rmsd) of 0.31 Å for the C^α^ atoms of residues 12–469 of the respective L subunits and 0.38 Å for the 123 C^α^ atoms of the respective S subunits. Only very subtle deviations in the main chain traces are evident suggesting that the presence of ABA does not cause a significant conformational change, at least at the lower occupancies in these crystals.

### (+)-ABA is predicted to bind to the non-activated Rubisco active site

**Fig 7:**
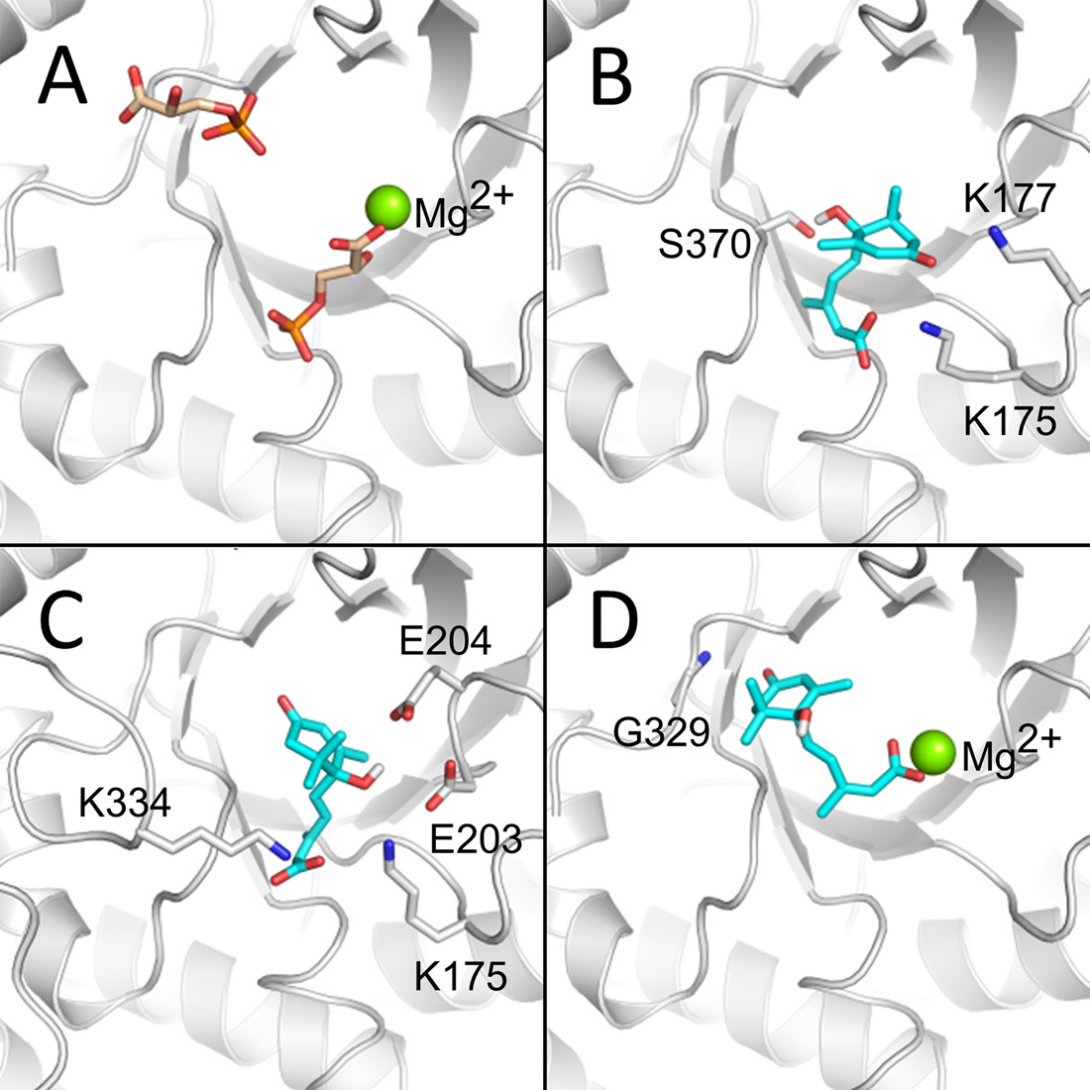
Predicted ABA binding to the spinach Rubisco active site. A) The Rubisco structure was previously solved in the presence of its two 3PGA product molecules (tan sticks with red oxygen atoms and orange phosphorus atoms), which may be used to define the protein’s active site (PDB ID: 1AA1). Small molecule docking of (+)-ABA (cyan sticks with red oxygen atoms) into B) the open, non-activated; C) closed, non-activated (PDB ID: 1RCX); and D) open, activated Rubisco active site, demonstrated the potential for competitive inhibition in a variety of enzyme states. In both non-activated enzyme forms, (+)-ABA was docked to the same location as the 3PGA molecule proximal to Mg2+ (PDB ID: 1AA1, residue 3PG 447) and was stabilized by hydrogen bonding to basic and acid residues (gray sticks) in Rubisco loops. In the activated enzyme form, (+)-ABA was docked to the region between the two 3PGA molecules, making ionic interactions with the Mg2+ cofactor (green spheres).

In the absence of diffraction-quality RuBP-free Rubisco crystals, small molecule docking was applied to assess (+)-ABA binding to the active site. This docking was performed using the 3PGA-extracted, activated, open-Rubisco structure (PDB ID: 1AA1); a 3PGA- and Mg^2+^-extracted, non-carbamoylated K201 version of the same open conformation structure to mimic a non-activated state; and RuBP-extracted, non-activated Rubisco in a closed conformation (PDB ID: 1RCX; Fig 7A). In the non-activated states, (+)-ABA was docked with highest affinity to the same location as the Mg^2+^-proximal 3PGA molecule (Fig 7B and 7C). These docked conformers identified a single orientation of the (+)-ABA aliphatic tail and terminal carboxylate with several, high affinity orientations of the cyclohexanone ring system. In both cases, (+)-ABA had the potential to make hydrogen bonds with K175, K177, D203 and E204. Additionally, (+)-ABA docked in the closed state had the potential to make additional contacts with loops comprising the lid that closes the active site (including residues in the regions of H327-E338 and A378-V385 (Fig 7C). In contrast, (+)-ABA was docked with only moderate affinity to the activated Rubisco active site in an orientation between the 3PGA molecules such that the carboxylate group coordinates the Mg^2+^ cofactor (Fig 7D). ABA could not be docked into the activated, closed Rubisco, due to strict occlusion of the binding site by Lys201 and Mg^2+^. Overall, these results support a model in which (+)-ABA binds preferentially to the non-activated Rubisco active site and elicits closure similar to the state observed upon RuBP binding, thereby stabilizing the interaction and the inhibition of the non-activated enzyme.

## Discussion

### Inhibition of Rubisco

To date, known inhibitors of Rubisco have been shown to exert their effects through inhibition of carbamoylation/activation (e.g. D-xylulose 1,5-bisphosphate, ribose 5-phosphate) or inhibition of subsequent carboxylation/catalysis (e.g. 2-carboxy D-arabinitol 1 –phosphate) [12]. In either case the inhibitors have been shown to bind in direct proximity to the catalytic site and serve as competitive inhibitors, directly limiting substrate binding and/or the relevant catalytic reaction. Indeed RuBP itself acts as an *in vivo* negative effector in the dark, when it forms a comparatively stable non-activated Rubisco-RuBP complex (*K*_D_ = 21 nM) relative to the activated Rubisco-RuBP complex (*K*_m_ = 21 uM) [13]. Rubisco activase is known to remove RuBP and other inhibitors from the catalytic site to enable carbon fixation during daylight [17]. Positive effectors on the other hand have been proposed to function through both allosteric sites (e.g. 6-phosphogluconate and fructose 1,6-bisphosphate [16]; orthophosphate [14]) and more recently through direct interaction with the catalytic site (e.g. NADPH, 6-phosphagluconate [15]). In the latter instance, the catalytic site is stabilized in an open conformation that promotes activation and substrate binding. Together these previous findings highlight that a wide range of mechanisms can regulate Rubisco and that the effect of a given metabolite may depend on the activation state and catalytic site conformation of the Rubisco enzyme at the time of interaction.

### ABA interactions with Rubisco

Now, the possible existence of multiple ABA-binding sites on Rubisco is raised. On the one hand is a high affinity site, characterized by the nM binding of ABA to non-activated, non-RuBP bound Rubisco (i.e. radiolabelling (Fig 3A)) and *in silico* docking data (Fig 7). On the other hand is a lower occupancy binding site of ABA, observed in a non-activated, RuBP-bound crystal structure (Fig 6). Together these data suggest the possibility of two unique ABA binding sites that exist on different regions of the enzyme. This is supported by the high stoichiometry reported for ABA binding to Rubisco by ITC (n = 19), which is not far off the expected 16 if two sites are filled on each of the 8 L subunits in the functional complex (Fig 4). As well observation of two different effects of (+)-ABA on Rubisco functionality including a weak non-competitive inhibition of the catalytic activity and a stronger, competitive inhibition of Rubisco activation at the active site (Fig 5), would also correlate with two distinct binding sites.

While not conclusively demonstrated, it is tempting to account for the weak, non-competitive, inhibition of Rubisco catalysis by the binding of ABA to the crystallographically identified low occupancy binding site at the location distal from the catalytic site. Indeed, the location of this site suggests two possible mechanisms of non-competitive or allosteric inhibition. Binding of the negatively-charged ABA molecule in proximity to the latch site might cause a slight destabilization of the positively-charged latch site, weakening the closed-activated conformation of Rubisco (Fig 6A). However, mutations of some of the latch site residues to Ala (causing a significant change in the overall positive character of the latch site) had no inhibitory effect on Rubisco activity, in apparent contradiction to this first mechanism [14]. Alternatively, the binding of ABA to this site, immediately adjacent to the loop involved in binding to Rubisco activase (residues 90–97), might place ABA in a position to interfere with the binding/activity of Rubisco activase [6]. However, Rubisco activase was not a component of the assay described herein and thus should not be relevant to the inhibition of activity observed. Overall, the crystallographic data yields a negative result, showing that there is no high affinity-binding site for ABA on RuBP-bound Rubisco. What the situation is in the absence of RuBP awaits the successful growth of RuBP-free, ABA-bound Rubisco crystals.

At the same time, one might also like to account for the more potent and competitive inhibition of Rubisco activation by the binding of ABA to the high-affinity ABA binding site detected by radiolabelled binding assays. Supporting this is the fact that both the high-affinity *K*_D_ of binding and the K_i_ for Rubisco activation were measured on the same non-activated Rubisco-ABA complex. This hypothesis would also fit a preferential binding mechanism that was proposed previously, wherein positive effectors of activation bind more tightly to the activated enzyme, while negative effectors (like RuBP) bind more tightly to the non-activated form [52]. Applying this here, ABA which binds strongly to the non-activated form, would be expected to act as an inhibitor of Rubisco activation, as observed. This possibility is further supported by subtle distortions of the electron density in the RuBP binding site and *in silico* predictions showing high affinity docking of (+)-ABA to a closed-conformation of the non-activated Rubisco (Fig 7). While consistent with the obtained binding data, the possibility of a high affinity ABA-binding site in the RuBP binding pocket raises the question of why there is virtually no clear veidence of binding of ABA at this site in the crystallographic electron density. Given comparable binding constants, one would expect RuBP and ABA to compete for the binding site. Whether RuBP has some structural advantage beyond affinity, possibly associated with loop 6 closure, leading to reduced ABA occupancy despite its high affinity, remains to be determined.

Ultimately, while interactions between ABA and Rubisco are identified, conclusive evidence of the mechanism of (+)-ABA inhibition of Rubisco activation/catalysis remains to be elucidated.

### Physiological relevance

Physiological relevance of any action of (+)-ABA on Rubisco must also be considered. Concentrations of Rubisco catalytic sites in the chloroplast are estimated to be 5 mM [6], while ABA has been reported to accumulate up to levels as high as ~ 0.45 μM in the chloroplast stroma of guard cells and 0.035 μM in the chloroplast of mesophyll cells in un-stressed leaves [58]. In both cell types, these values were higher than the ABA concentrations detected in the bulk cytoplasm. Total plant ABA levels have been shown to increase between 3–30 fold upon stress induction [59], which would suggest a possible maximum of 12–15 μM maximum in the chloroplast. Thus, most likely, the weak inhibition (*K*_i_ = 2.1 mM) of Rubisco catalysis is not biologically relevant, nor the associated, low occupancy surface binding site for (+)-ABA, determined crystallographically herein. However, the high affinity site on the non-activated conformation, with a binding *K*_D_ of 5 nM, could be relevant, particularly in the context of inhibition of Rubisco activation (*K*_i_ 130 μM).

In this context it is also important to note a previous report, looking at carbon assimilation and stomatal function in WT and ABA-deficient tomatoes (with bulk leaf ABA concentrations differing by more than 50%) [60]. This work showed statistically identical carbon assimilation rates as a function of CO_2_ pressure and exogenously added ABA, in both plant types, and concluded no effect of ABA on plant photosynthetic capacity. However a decrease in the RuBP regeneration at high CO_2_ levels for all plants and treatment conditions was also reported. With, any possibility of this effect being associated with the competing oxygenase activity of Rubisco eliminated by the controlled CO_2_ environment, the authors state that ‘some unknown factor is limiting photosynthesis at high CO_2_ levels’ [60]. Our own closer examination of this data does show a non-statistically significant reduction in carbon assimilation for ABA treated plants only, at high light and high CO_2_; but the data is ultimately inconclusive about any role for inhibition of photosynthesis/Rubisco by ABA.

## Conclusions

A negative regulatory role for ABA with respect to Rubisco function would be consistent with ABA’s documented role in shifting priority from carbon assimilation to water conservation during drought stress. The biochemical findings reported here support the possibility of two ABA binding sites on Rubisco including distinct low and high affinity sites. We propose that the low occupancy binding site and possible associated weak non-competitive inhibition of catalysis, may not be relevant *in vivo*, but suggest that the high affinity site may allow ABA to act as a negative effector of Rubisco activation. While much remains enigmatic, these findings represent the first evidence of a direct interaction between stress response factors and photosynthetic enzymes, providing interesting lines for future investigation.

## Supporting Information

S1 Fig SDS-PAGE of Rubisco. Spinach Rubisco (Sigma-Aldrich) was analyzed on a 15% SDS-PAGE. A PageRuler Plus Prestained Protein Ladder (Thermo Scientific) was run on lane 1. Approximately 20 μg and 40 μg of spinach Rubisco were run on lanes 2 and 3, respectively. The large subunit and small subunit of Rubisco are indicated by L and S, respectively.
(TIF)

S2 Fig MS/MS ion search results (Mascot) of the digested gel regions. Protein fractions were eluted by streptavidin—Sepharose affinity columns, desalted, concentrated using AmiconTM Ultrafree centrifugal filters (Millipore), and visualized using a FOCUS-FAST silver-stain kit. The A (top panel), B (Middle panel) and C (lower panel) bands were excised and analysed by LC-S/MS and Mascot ion search methods as described in the Experimental section of the main paper.
(TIF)

S3 Fig Effect of ABA on initial rate versus equilibrium rate of spinach Rubisco catalysis. Non-activated Rubisco was preincubated with no (+)-ABA (blue lines) or 100–250 μM (+)-ABA (red lines) and the reaction initiated by the addition of Mg^2+^, 7mM NaHCO_3_ and a solution of coupled reactions including NADH as a final substrate for monitoring reaction progress, as described in the methods and materials. The Left panel shows the effect of ABA on the initial rate up to 2 minutes into the reaction, which is representative of Rubisco activation. The Right panel shows the same reaction from 3–5 minutes after initiation, when the Rubisco is all activated and an equilibrium state of catalysis is reached. Each line is an average of at least n = 3.
(TIF)
