# Supplementary figures and images for: Identification of Interactions between Abscisic Acid and Ribulose-1,5-Bisphosphate Carboxylase/Oxygenase
Source: PLoS One. 2015 Jul 21;10(7):e0133033. doi: 10.1371/journal.pone.0133033 (PMC4510133; doi:10.1371/journal.pone.0133033)

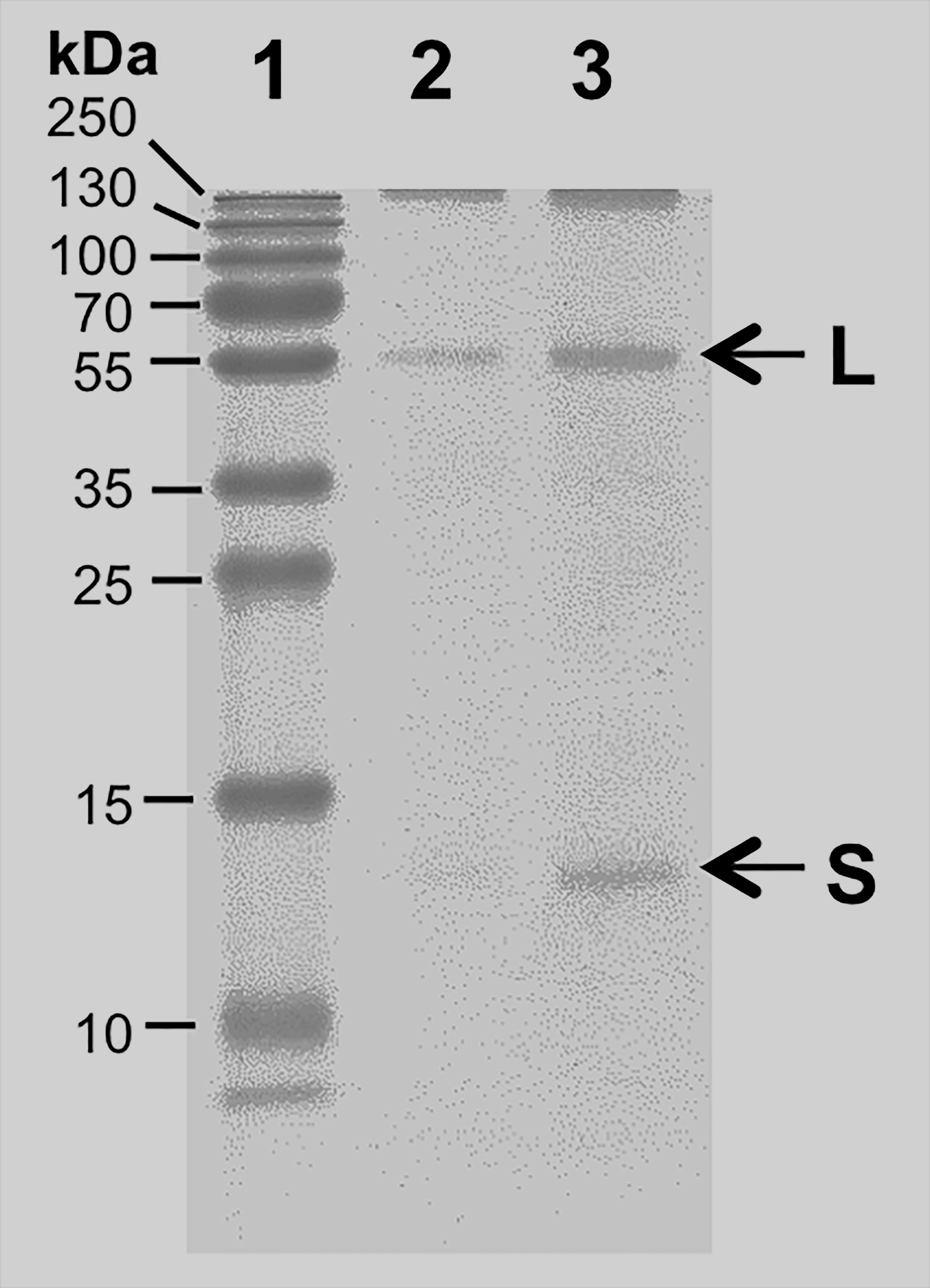

Supplement: S1 Fig — Spinach Rubisco (Sigma-Aldrich) was analyzed on a 15% SDS-PAGE. A PageRuler Plus Prestained Protein Ladder (Thermo Scientific) was run on lane 1. Approximately 20 μg and 40 μg of spinach Rubisco were run on lanes 2 and 3, respectively. The large subunit and small subunit of Rubisco are indicated by L and S, respectively. (TIF) [file pone.0133033.s001.tif]

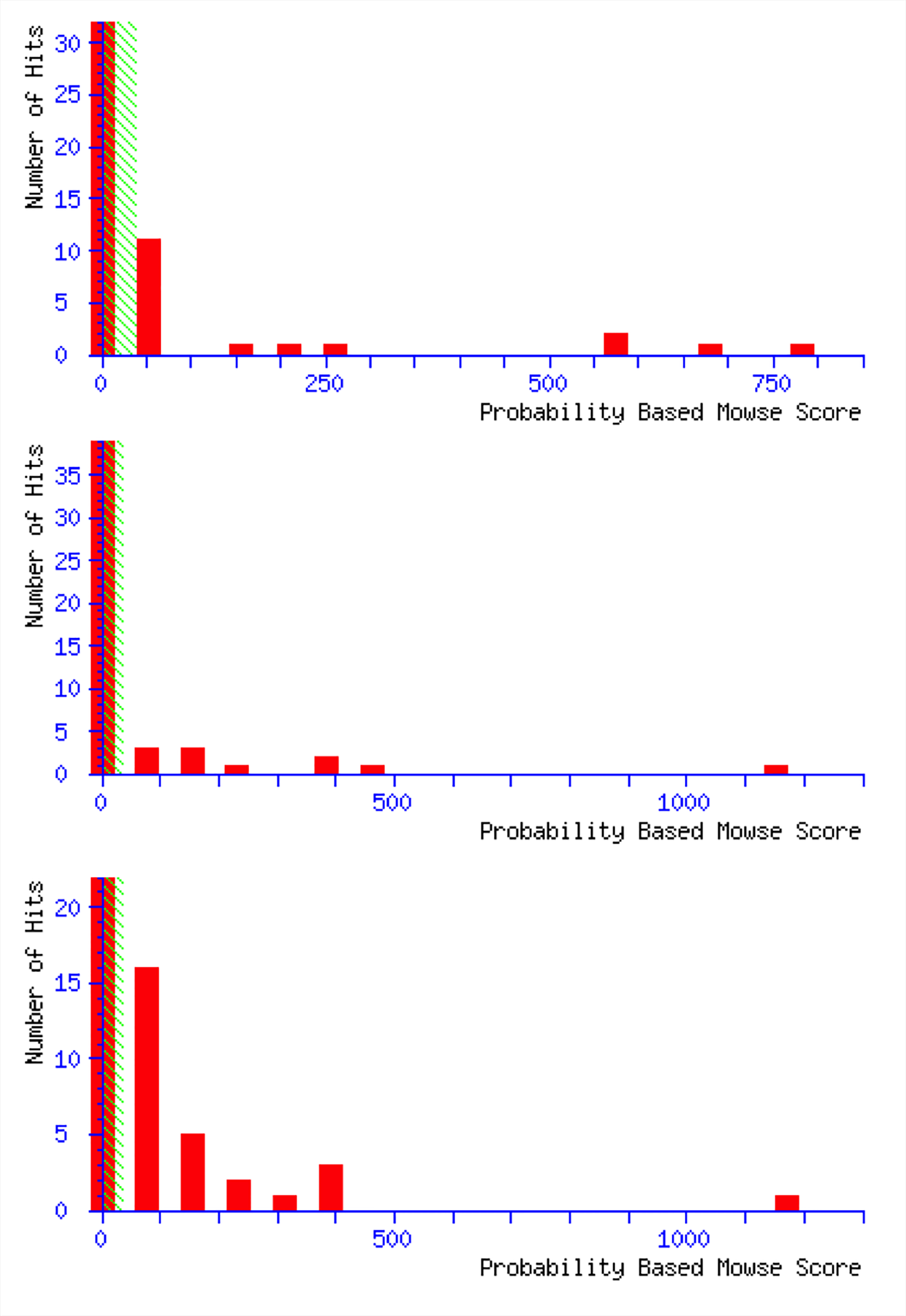

Supplement: S2 Fig — Protein fractions were eluted by streptavidin—Sepharose affinity columns, desalted, concentrated using AmiconTM Ultrafree centrifugal filters (Millipore), and visualized using a FOCUS-FAST silver-stain kit. The A (top panel), B (Middle panel) and C (lower panel) bands were excised and analysed by LC-S/MS and Mascot ion search methods as described in the Experimental section of the main paper. (TIF) [file pone.0133033.s002.tif]

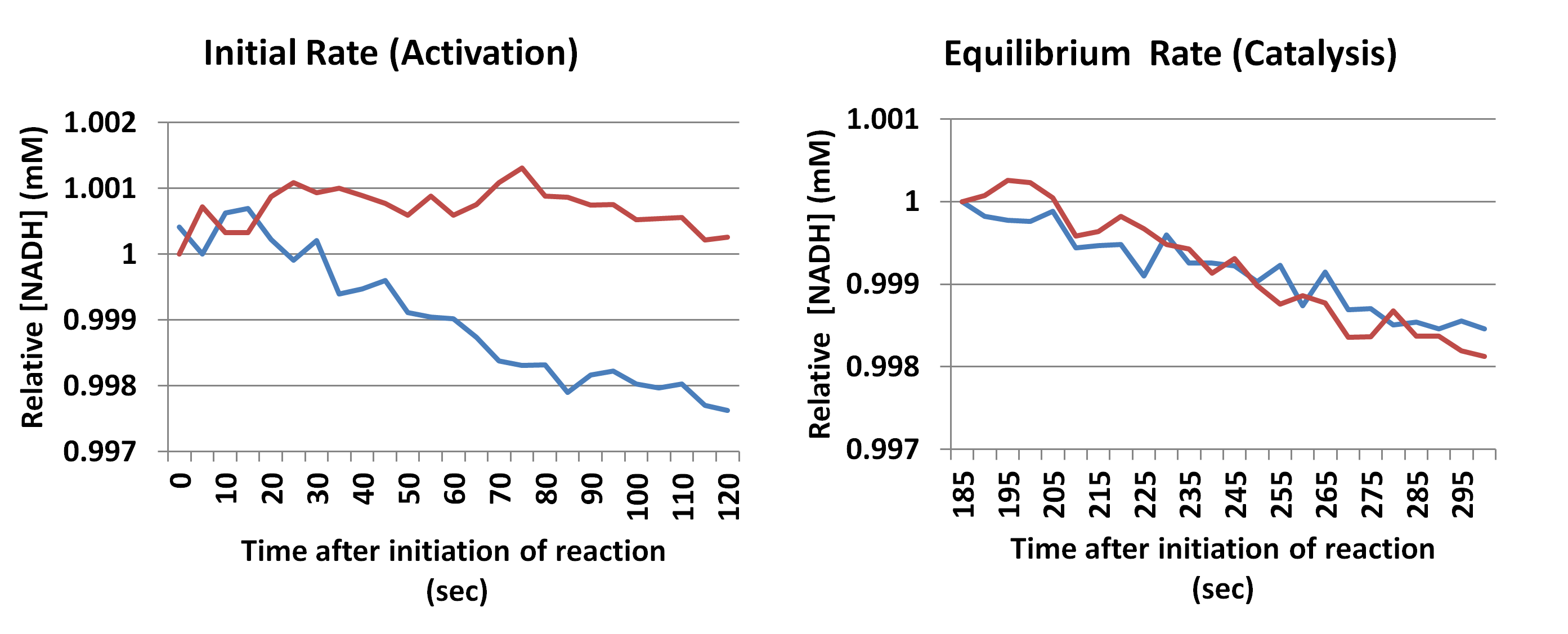

Supplement: S3 Fig — Non-activated Rubisco was preincubated with no (+)-ABA (blue lines) or 100–250 μM (+)-ABA (red lines) and the reaction initiated by the addition of Mg2+, 7mM NaHCO3 and a solution of coupled reactions including NADH as a final substrate for monitoring reaction progress, as described in the methods and materials. The Left panel shows the effect of ABA on the initial rate up to 2 minutes into the reaction, which is representative of Rubisco activation. The Right panel shows the same reaction from 3–5 minutes after initiation, when the Rubisco is all activated and an equilibrium state of catalysis is reached. Each line is an average of at least n = 3. (TIF) [file pone.0133033.s003.tif]
